# Reversible Charge‐Polarity Control for Multioperation‐Mode Transistors Based on van der Waals Heterostructures

**DOI:** 10.1002/advs.202106016

**Published:** 2022-07-13

**Authors:** Ciao‐Fen Chen, Shih‐Hsien Yang, Che‐Yi Lin, Mu‐Pai Lee, Meng‐Yu Tsai, Feng‐Shou Yang, Yuan‐Ming Chang, Mengjiao Li, Ko‐Chun Lee, Keiji Ueno, Yumeng Shi, Chen‐Hsin Lien, Wen‐Wei Wu, Po‐Wen Chiu, Wenwu Li, Shun‐Tsung Lo, Yen‐Fu Lin

**Affiliations:** ^1^ Department of Electrophysics and Center for Emergent Functional Matter Science (CEFMS) National Yang Ming Chiao Tung University Hsinchu 30010 Taiwan; ^2^ Department of Physics National Chung Hsing University Taichung 40227 Taiwan; ^3^ International Collaborative Laboratory of 2D Materials for Optoelectronics Science and Technology (Ministry of Education) Engineering Technology Research Center for 2D Material Information Functional Devices and Systems (Guangdong Province) Institute of Microscale Optoelectronics Shenzhen University Shenzhen 518060 China; ^4^ Department of Materials Science and Engineering National Yang Ming Chiao Tung University Hsinchu 300 Taiwan; ^5^ Institute of Electronics Engineering National Tsing Hua University Hsinchu 30013 Taiwan; ^6^ Department of Chemistry Graduate School of Science and Engineering Saitama University Saitama 338–8570 Japan; ^7^ Center for the Intelligent Semiconductor Nano‐system Technology Research National Yang Ming Chiao Tung University Hsinchu 300 Taiwan; ^8^ Shanghai Frontiers Science Research Base of Intelligent Optoelectronics and Perception, Zhangjiang Fudan International Innovation Center Institute of Optoelectronics Department of Materials Science Fudan University Shanghai 200433 China; ^9^ Department of Materials Science and Engineering Institute of Nanoscience i‐Center for Advanced Science and Technology (i‐CAST) National Chung Hsing University Taichung 40227 Taiwan

**Keywords:** charge‐polarity control, MoTe_2_, multioperation‐mode transistors, SnS_2_, van der Waals heterostructures

## Abstract

Van der Waals (vdW) heterostructures—in which layered materials are purposely selected to assemble with each other—allow unusual properties and different phenomena to be combined and multifunctional electronics to be created, opening a new chapter for the spread of internet‐of‐things applications. Here, an O_2_‐ultrasensitive MoTe_2_ material and an O_2_‐insensitive SnS_2_ material are integrated to form a vdW heterostructure, allowing the realization of charge‐polarity control for multioperation‐mode transistors through a simple and effective rapid thermal annealing strategy under dry‐air and vacuum conditions. The charge‐polarity control (i.e., doping and de‐doping processes), which arises owing to the interaction between O_2_ adsorption/desorption and tellurium defects at the MoTe_2_ surface, means that the MoTe_2_/SnS_2_ heterostructure transistors can reversibly change between unipolar, ambipolar, and anti‐ambipolar transfer characteristics. Based on the dynamic control of the charge‐polarity properties, an inverter, output polarity controllable amplifier, p‐n diode, and ternary‐state logics (NMIN and NMAX gates) are demonstrated, which inspire the development of reversibly multifunctional devices and indicates the potential of 2D materials.

## Graphical abstract

Although various van der Waals stacking systems for preparing multifunctional devices are reported, their complexity in fabrication and/or inflexibility in reversible operation limit the possibility of multifunctional integrations and the requirement of the fast‐growing internet of things paradigm. Here, a simple and effective rapid temperature annealing strategy for reversibly controlling and optimizing the electronic properties of MoTe_2_‐based heterostructure electronics is proposed/realized.

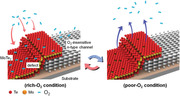

## 1 Introduction

With the continuous scaling down of electronics and the urgent need to handle data explosion, the internet of things (IoTs) has risen in use and popularity in the past decade. The IoTs comprise widely distributed devices with multifunctionality that are interconnected, providing real‐time communication of the scope of what we want to know and what we want to do.^[^ ^1^, ^2^, ^3^ ^]^ The new era of the IoTs not only highlights a productive new avenue for a whole generation of information devices but also provides an opportunity to surpass the limits of Moore's law. A range of novel 2D materials has been actively explored for More Moore and More‐than‐Moore applications because of their unique structures and optoelectronic properties.^[^ ^4^, ^5^, ^6^ ^]^ By vertically or/and laterally assembling the 2D materials, its integration of artificial van der Waals (vdW) heterostructures can be further used to create diverse devices including tunneling transistors,^[^ ^7^, ^8^ ^]^ atomically p‐n junctions,^[^ ^9^, ^10^, ^11^, ^12^ ^]^ light‐emitting diodes,^[^ ^13^ ^]^ reconfigurable field‐effect transistors,^[^ ^14^, ^15^ ^]^ nonvolatile memories,^[^ ^16^, ^17^ ^]^ and even multifunctional devices.^[^ ^18^, ^19^, ^20^, ^21^, ^22^, ^23^, ^24^ ^]^ These atomically vdW heterostructures‐based systems, possessing ultra‐low‐power, low‐loss, and energy‐efficient active features, fully satisfy the requirement of the fast‐growing IoTs paradigm.

The assembled vdW heterostructures, which do not suffer from impurities or interfacial defects, have high‐quality interfaces and are highly suited to use in state‐of‐the‐art electronics with desired multi‐functionalities.^[^ ^25^, ^26^ ^]^ For example, in 2015, a band‐to‐band tunneling transistor in a MoS_2_/WSe_2_ vdW heterostructure was made using a dual‐gate device architecture.^[^ ^18^ ^]^ Under different gate‐voltage (*V*_g_) modulations, the vdW system behaved as either an Esaki diode, a backward diode, or a forward rectifying diode, making the device highly versatile. In 2017, a fine band‐structure alignment using narrow‐bandgap black phosphorus (BP) and large‐bandgap MoS_2_ stacking was achieved, in which a large current rectification ratio of 10^6^ and a high on–off current ratio up to 10^7^ were realized by integrating both the high‐performance diode and transistor in a single device.^[^ ^19^ ^]^ A ternary inverter can be further achieved by matching the partition load and band structure in the vertical heterostructure. In the same year, because the charge‐carrier injection can be switched between tunneling and thermal activation under different bias polarities, a multifunctional device, including a diode, transistor, photodetector, and multi‐state memory, was reported in an asymmetric MoTe_2_/MoS_2_‐based vdW heterostructure.^[^ ^20^ ^]^ A similar asymmetric configuration combined with a floating‐gate charge layer was also been adopted in WSe_2_/hexagonal boron nitride/graphene heterostructures for multi‐purpose optoelectronic applications.^[^ ^22^ ^]^ In addition, in BP/ReSe_2_ heterostructures, the tunability of diverse current‐transport characteristics was demonstrated using the variation of the BP layer thickness.^[^ ^27^ ^]^ To date, most of the above‐related approaches for preparing multifunctional devices have been limited by either complexity in fabrication technology and/or inflexibility in reversible operation, which significantly limit the possibility of multifunctional integrations.

Precisely controlling the conduction behavior, such as conductive polarities or doping degrees, is the key to achieving practical applications for vdW heterostructures.^[^ ^23^, ^28^, ^29^, ^30^, ^31^ ^]^ In general, after choosing specified 2D materials, the vdW devices will exhibit unchanged conduction behaviors, because the conduction behaviors as well as band structure alignments, directly depend on the nature and corresponding thickness of the selected 2D materials. A convertible conducting channel in a single device based on a vdW system, which allows for higher integration and compatibility, is still lacking. Herein, we propose a new and simple strategy for dynamically controlling and optimizing the electronic properties of vdW heterostructures‐based electronics, and then realize their multifunctional applications in a “single device.” MoTe_2_ has been proved to be ultrasensitive to O_2_.^[^ ^32^, ^33^, ^34^, ^35^, ^36^ ^]^ Charge carriers of MoTe_2_ flakes could be easily modulated, which is primarily attributed to the amount of O_2_ adsorptions on the MoTe_2_ surface, particularly at its tellurium defects. Such the adsorption process is reversible when MoTe_2_ flakes are stored in a reversed‐O_2_ condition. Therefore, in this study, through the rapid thermal annealing (RTA) strategy between dry‐air (80% N_2_, and 20% O_2_, 1 atm) and vacuum conditions, a tunable charge‐polarity device based on MoTe_2_/SnS_2_ vdW heterostructures were fabricated. Compared with MoTe_2_, the electrical properties in SnS_2_ with a suitable thickness exhibit relative stability in different O_2_ concentrations. The simple RTA strategy in different conditions enables precise tuning of the doping level and effective modulation of the transport behavior in the MoTe_2_ flakes in a large dynamic range, thus allowing the MoTe_2_/SnS_2_ vdW heterostructure to be reversibly switched between unipolar, ambipolar, and anti‐ambipolar‐dominated transistors. Finally, the flexible multioperation modes of logic‐circuit applications, for example, inverter, p‐n diode, output polarity controllable (OPC) amplifiers, and ternary‐state NMIN and NMAX logics can be precisely engineered. Our proposed strategy for multioperation‐mode transistors is highly promising in the prospective of future electronics, as well as the growing IoTs generation.

Bulk 2H‐phase MoTe_2_ is an indirect bang gap material, with a band gap in the range of 0.6–1 eV.^[^ ^37^ ^]^ At the beginning of research on layered MoTe_2_ transistors, ambipolar behavior in the charge polarity has been revealed.^[^ ^38^ ^]^ Subsequently, it has been demonstrated that oxygen present in the environment can significantly alter the electrical properties of layered MoTe_2_ devices. Such related research can be traced back to 2015.^[^ ^32^, ^33^ ^]^ The presence of tellurium defects and the functionalization of these defect sites with O_2_ molecules strongly dictate the electronic structure and create deep or shallow states with the optical band gap. Moreover, by varying the vacuum level during the RTA,^[^ ^34^, ^36^, ^39^, ^40^ ^]^ electrothermal doping process,^[^ ^41^ ^]^ and scanning visible‐light method,^[^ ^42^, ^43^ ^]^ the transfer characteristics of layered MoTe_2_ transistors can distinctly shift to show different polarities between unipolar‐like and symmetric ambipolar behaviors owing to the physical or chemical adsorption and dissociation of O_2_ associated with the energy level of these strategies.

**Figure 1:**
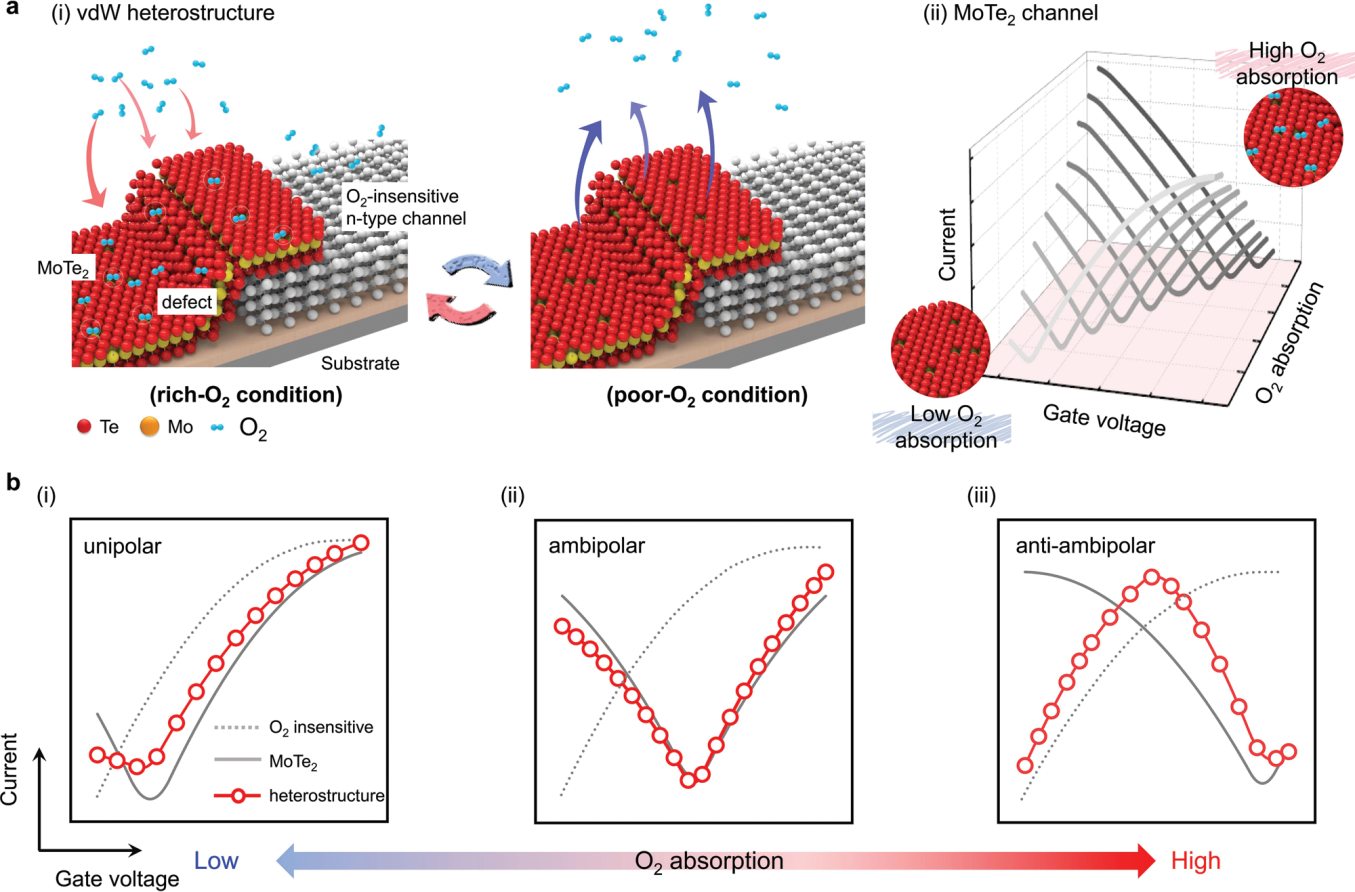
a) Simplified principle of reversible charge‐polarity control for a multioperation‐mode transistor. i) Schematic illustration of oxygen adsorption and desorption processes on the surface of a MoTe_2_‐based vdW heterostructure. Owing to the presence of Te vacancies on exfoliated MoTe_2_ surfaces, the electrical properties of the MoTe_2_‐based vdW heterostructure were highly sensitive to different O_2_ concentration environments. ii) Corresponding transfer characteristics of a MoTe_2_ channel with different degrees of oxygen adsorption. b) Schematic diagrams of the transfer‐curve shift for a MoTe_2_‐based vdW heterostructure. Under different degrees of O_2_ adsorption, the vdW heterostructure shows i) unipolar, ii) ambipolar, and iii) anti‐ambipolar charge polarity.

Based on the O_2_‐ultrasensitive MoTe_2_ surface, **Figure** **1**a shows a schematic diagram of reversible charge‐polarity control for a multioperation‐mode vdW heterostructure, in which two layered materials, MoTe_2_ and an O_2_‐insensitive n‐type flake, were selected to integrate with a vertical stack to form a vdW transistor. First, we considered that in ambient conditions (i.e., relatively high O_2_ concentration), the O_2_ vapor naturally adsorbs on the MoTe_2_ surface, in particular, at the tellurium defects, where charge carriers for MoTe_2_ would be trapped, and then its polarity becomes hole dominated. An effective p‐type doing of MoTe_2_ was hence developed, as shown in the left‐hand panel of Figure 1ai. However, the adsorbed O_2_ with low adsorption energy on the MoTe_2_ surface would potentially be removed when the MoTe_2_‐based vdW heterostructure was put in vacuum (i.e., relatively low O_2_ concentration) condition for a period of time or a given additional energy. Such desorption of O_2_ could release charge carriers again back to the MoTe_2_, leading to n‐type doping of the vdW heterostructure, as shown in the right‐hand panel of Figure 1ai. This process of O_2_ adsorption/desorption has been proven to be controlled and reversible, freely creating p/n charge polarity in MoTe_2_. A typical variation of transfer characteristics of a MoTe_2_ channel is illustrated in Figure 1aii for different degrees of O_2_ adsorption. Schematic diagrams of the transfer curve shift for the MoTe_2_‐based vdW heterostructure with increasing adsorbed O_2_ concentration, are shown in Figure 1b for i) unipolar, ii) ambipolar, and iii) anti‐ambipolar‐dominated charge‐polarity transistors. Thus, through O_2_ adsorption‐evacuation cycles, the inflexibility in charge polarity for typical vdW heterostructures can be broken, achieving multioperation‐mode transistors.

**Figure 2:**
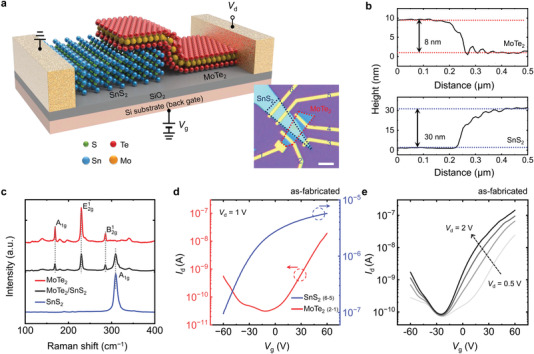
a) Schematic diagram of a van der Waals stacked MoTe_2_/SnS_2_ heterostructure transistor on a SiO_2_/Si substrate. The inset shows an optical image of the as‐fabricated transistor, where a few‐layer MoTe_2_ flake was exfoliated on top of an SnS_2_ flake. The electrode contact on SnS_2_ was grounded while a source–drain voltage was applied to the MoTe_2_ contact. The scale bar represents 3 µm. The labels for each electrode are marked in the optical image. b) Height profiles showing the thickness of the MoTe_2_ (top) and SnS_2_ (bottom) flakes. c) Raman spectra collected from the different regions corresponding to MoTe_2_, SnS_2_, and the heterostructure. d) Transfer characteristic curves of the as‐fabricated MoTe_2_ and SnS_2_ transistors at *V*_d_ = 1 V before annealing treatment. e) Transfer characteristic curves of the as‐fabricated MoTe_2_/SnS_2_ heterostructure at different *V*_d_ values before annealing treatment.

To realize the concept of reversible charge‐polarity control for multioperation‐mode transistors based on vdW heterostructures outlined in Figure 1, vertically stacked MoTe_2_/SnS_2_ heterostructure transistors were fabricated. A schematic diagram of the structural configuration of a MoTe_2_/SnS_2_ heterostructure with the circuit layout is shown in **Figure** **2**a. To enhance the adsorbed/removed O_2_ effect on the MoTe_2_ surface, a few‐layered MoTe_2_ flake was intentionally stacked on top of the SnS_2_ flake. Ti/Au films were used as metallic electrodes to optimize the electrical properties and adhesion. The electrode contact on SnS_2_ was grounded, while a source–drain voltage (*V*_d_) was applied on the MoTe_2_. The inset of Figure 2a shows an optical image of the as‐fabricated MoTe_2_/SnS_2_ vdW heterostructure transistor. Atomic force microscopy (AFM) was performed to measure the thickness of the MoTe_2_ and SnS_2_ flakes. The corresponding height profiles (Figure 2b) show that the thicknesses of the MoTe_2_ and SnS_2_ flakes were ≈8 and 30 nm, respectively. An AFM image of the vdW heterostructure is shown in Figure S1, Supporting Information. Raman scattering measurement was then carried out on different channel areas of the as‐fabricated transistor and the results are shown in Figure 2c. The peak of A^1^_g_ at 172 cm^−1^, E^1^_2g_ at 232 cm^−1^, and B^1^_2g_ at 291 cm^−1^ correspond to MoTe_2_ (red line), while the Raman spectrum of the SnS_2_ shows an A^1^_g_ peak at 314 cm^−1^ (blue line). All of these peak positions are consistent with previous reports.^[^ ^44^ ^]^ In the heterostructure region (black line), the peaks show contributions from both materials, indicating the presence of two distinct materials. Figure 2d further shows transfer characteristics (*I*_d_−*V*_g_) of the as‐fabricated MoTe_2_ and SnS_2_ transistors at *V*_d_ = 1 V before any annealing treatment. The transfer in the as‐fabricated MoTe_2_ is dominated by n‐ and p‐type carriers under positive and negative gate biases, respectively, resulting in ambipolar behavior (red curve), while the as‐fabricated SnS_2_ shows n‐type transport throughout the entire scan from −60 to 60 V (blue curve). Unlike other anti‐ambipolar heterostructures reported in previous work,^[^ ^45^ ^]^ our as‐fabricated MoTe_2_/SnS_2_ heterostructure exhibits electron‐dominated ambipolar transfer characteristics at different *V*_d_ values before annealing, as shown in Figure 2e, primarily because the hole doping of the as‐fabricated MoTe_2_ is far from its balanced level. The corresponding output characteristic of the heterostructure has also been monitored to display no rectification, indicating the formation of good contacts at the semiconductor‐metal interfaces (not shown here).

**Figure 3:**
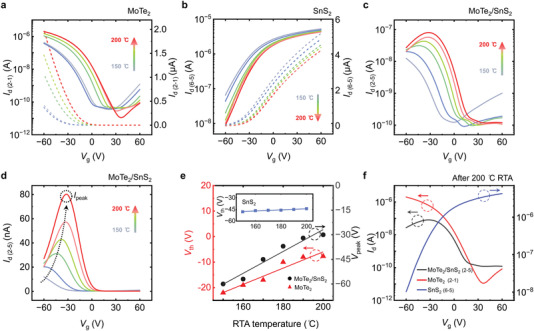
Transfer characteristic curves for the a) MoTe_2_ and b) SnS_2_ transistors on semilog (solid line) and linear (dashed line) scales. c,d) Transfer characteristic curves of the MoTe_2_/SnS_2_ vdW heterostructure on semilog and linear scales, respectively. The curves in different colors correspond to different annealing temperatures from 150 to 200 °C under dry‐air conditions during the RTA process. The height of maximum *I*_d_ values is marked as *I*_peak_ in (d). e) Threshold voltage (*V*_th_) of the MoTe_2_ transistor (red) and the voltage position of the *I*_peak_ of the MoTe_2_/SnS_2_ heterostructure (black) as a function of annealing temperature in dry‐air conditions. The inset shows the *V*_th_ of the SnS_2_ transistor as a function of the annealing temperature. f) Transfer characteristic curves of the MoTe_2_, SnS_2_, and MoTe_2_/SnS_2_ vdW heterostructure after 200 °C RTA treatment. All the conditions were measured at *V*_d_ = 1 V.

It has been demonstrated that the binding energy arising from the interaction between O_2_ (and/or H_2_O) and the layered MoTe_2_ surface is only in the range of 30–60 meV,^[^ ^32^ ^]^ which is comparable to room‐temperature energy and strongly implies that adsorbed molecules could be desorbed by suitable thermal energy. Hence, we deliberately adopted the RTA process to accelerate the O_2_ reaction under dry‐air and vacuum conditions, then developed a versatile O_2_ doping approach that enables highly effective and nonvolatile band modulation of the MoTe_2_‐based vdW heterostructures. Doping was done by applying the RTA process on the vdW transistor at a fixed temperature for 1 min under dry‐air conditions. The vdW transistor was then put into the probe station and a transfer curve was recorded to characterize its transport behavior. The transfer characteristics for different annealing temperatures from 150 to 200 °C are shown in **Figures** **3**a and 3b for the MoTe_2_ and SnS_2_ transistors, respectively. The solid and dashed lines are semilog and linear plots, respectively. It is noticed that no meaningful hysteresis loop was observed in the transfer curves, either activation condition exists or not. The magnitude of the change was qualitatively proportional to the annealing temperature. As shown in Figure 3a, the MoTe_2_ transistor transforms from having electron‐dominated ambipolar behavior before annealing to a highly doped unipolar p‐type transistor as the annealing temperature increases from 150 to 200 °C. In contrast, the SnS_2_ transistors underwent the same RTA treatment and showed very little response despite a gradual reduction in on‐state current (*I*_d_) from 5.2 to 4.2 µA at *V*_g_ = 60 V (see Figure 3b). Such a relatively weak response to the RTA process mainly results from a lower surface‐to‐volume ratio of the thicker SnS_2_ channels because the molecule adsorption/desorption is a surface effect. The thickness evidence is shown in Figure S2, Supporting Information. It was found that the *I*_d_ values at a fixed *V*_g_ condition for a thin SnS_2_ transistor significantly dropped by two orders of magnitude after the RTA process, as shown in Figure S2a,b, Supporting Information, which provides direct evidence to support our expectation. Therefore, a thicker SnS_2_ flake was selected as an O_2_‐insensitive n‐type material to give reversible charge‐polarity vdW heterostructures. Here, to maintain a suitable current on–off modulation, the range of the SnS_2_ thickness was typically chosen from 20 to 50 nm for vdW p‐n stackings.

Figures 3c and 3d show the transfer characteristics of the MoTe_2_/SnS_2_ vdW heterostructure with different annealing temperatures from 150 to 200 °C under a dry‐air condition in semilog and linear scales, respectively. The vdW transistor evolved from having electron‐dominated ambipolar behavior before annealing (see Figure 2e) to highly balanced ambipolarity (gray line in Figure 3c), and then to anti‐ambipolar charge transport (red line in Figure 3c) as the annealing temperature increased from 150 to 200 °C. In the linear plot of the transfer curves, the height of maximum *I*_d_ values is marked as *I*_peak_ (see Figure 3d). As the annealing temperature increases, the *I*_peak_ gradually increases, bringing a more obvious anti‐ambipolar charge transport, as well as p‐type doping in the MoTe_2_. In addition, the positions of the corresponding *I*_peak_ (i.e., *V*_peak_) and threshold voltage (*V*_th_) for the MoTe_2_ and SnS_2_ transistors are displayed as a function of annealing temperature in Figure 3e. The *V*_peak_ and the *V*_th_ of MoTe_2_ increased linearly with the annealing temperature from 150 to 200 °C, whereas the SnS_2_ transistor maintained a *V*_th_ of around −45 V (inset), regardless of thermal treatment, suggesting again that the charge‐polarity change of the vdW transistor is attributed to the doped MoTe_2_. Through the RTA doping treatment, we were able to reconfigure the MoTe_2_ transistor to the desired properties for a suitable device application. The outcome in Figure 3f verifies the clear formation of the anti‐ambipolar vdW heterostructure at the junction of two opposite polarity transistors under 200 °C RTA treatment, in which the off‐state voltages around −60 and 36 V are displayed. It is emphasized that the maximum of applied *V*_d_ to the vdW heterostructure is not higher than 2 V, which makes no meaningful impact on Joule heating to alter the charge polarity. In short, using the simple RTA strategy, we can precisely control the transition from the electron‐dominated ambipolar to anti‐ambipolar states and accurately modulate both the *I*_peak_ height, as well as its position, which is critical for building more functional blocks using the vdW heterostructures.

**Figure 4:**
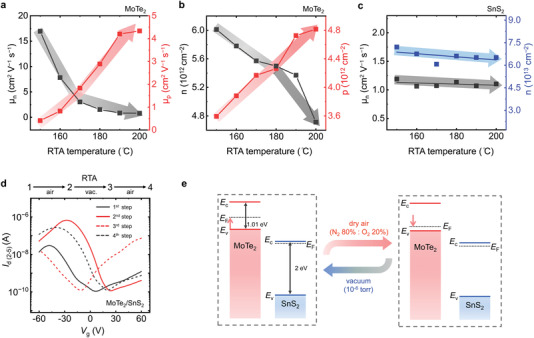
a) Mobilities and b) carrier concentrations for holes and electrons in the MoTe_2_ transistors as a function of annealing temperature in dry‐air conditions. c) Mobilities and carrier concentrations for electrons in the SnS_2_ transistors as a function of annealing temperature in dry‐air conditions. d) Reversibility of the RTA process, including cyclic exposure of the MoTe_2_/SnS_2_ vdW heterostructure to dry air and vacuum. Transfer properties of the vdW heterostructure before the RTA process (first step, solid black line). Transfer properties of the vdW heterostructure processed by the RTA process in dry air (second step, solid red line). Transfer features of the vdW heterostructure after the RTA process in a vacuum (third step, dashed red line) and dry air (fourth step, dashed black line). e) Corresponding band diagrams of the MoTe_2_/SnS_2_ vdW heterostructure during the cycle (i.e., dry air and vacuum) of the RTA process.

To visualize the polarity change process during the RTA treatment, the conventional equation *μ*_*n*,*p*_ = (*L*/*W*)*C_g_g_m_V_d_* was used, where *μ*_*e*,*h*_ is the field‐effect mobility for electrons and holes, *L*/*W* is the channel length‐to‐width ratio, *C*_g_ = 1.15 × 10^−8^ F cm^−2^ is the gate‐oxide capacitance per unit area for a 300‐nm SiO_2_ dielectric and $g_{m}=dI_{d}/dV_{d}$ is transconductance. The MoTe_2_ mobility for holes was found to increase by one order of magnitude from 0.4 to 4.3 cm^2^ V^−1^ s^−1^ at *V*_d_ = 1 V at the highest annealing temperature (i.e., 200 °C), while that for electrons decreased from 16.9 to 0.8 cm^2^ V^−1^ s^−1^ at *V*_d_ = 1 V, as depicted in **Figure** **4**a. Further taking the relation of *n*, *p* = (*I_d_* · (*L*/*W*))/(*qV_d_μ*_*n*,*p*_) into consideration, where *q* is the elementary charge, the corresponding carrier concentration (*p*) for holes increased by 1.4 times from 3.5 × 10^12^ to 4.3 × 10^12^ cm^−2^, while that (*n*) for electrons decreased by 1.2 times from 6 × 10^12^ down to 4.7 × 10^12^ cm^−2^, which provides consistent evidence for the adsorption of O_2_ molecules on the MoTe_2_ surface resulting in the down‐shifted Fermi level (*E*_F_) in MoTe_2_ (see Figure 4b). In contrast, both the mobility and carrier concentration for SnS_2_ remained constant to be 1.1 cm^2^ V^−1^ s^−1^ and 6.6 × 10^13^ cm^−2^, respectively, as a function of annealing temperature (Figure 4c), again confirming the O_2_‐insensitive properties of SnS_2_ at suitable flake thickness. In this work, we monitored more than ten devices treated using the same RTA strategy. There were unavoidable variations, such as doping mobility and carrier concentration for individual material or *I*_peak_ (*V*_peak_) position and on‐state current for anti‐ambipolar transfer characteristics due to differences in channel quality, geometry, and minute process‐to‐process deviations, but all devices behaved the same qualitatively in terms of reversibility between the ambipolar and anti‐ambipolar states and tunability of the peak position under the RTA treatment (see Figure S3, Supporting Information). In addition, the RTA treatment doping was bidirectional and reversible when the MoTe_2_/SnS_2_ vdW heterostructure was exposed cyclically between the dry‐air and vacuum conditions to allow O_2_ adsorption/desorption on the MoTe_2_ surface, as shown in Figure 4d. The corresponding reversibility for the MoTe_2_ and SnS_2_ transistors is shown in Figure S4, Supporting Information. Notice that the transfer curve of the vdW heterostructure can be back to the as‐fabricated case, like the result presented in Figure 1e, if the RTA temperature applied was higher than 200 °C and the device could withstand an extended treatment time. However, this introduced a greater possibility of damage to the 2D materials, which is not within the scope of this work and is not discussed here. Since the RTA improvement of contact resistance at the metal‐semiconductor interface^[^ ^46^ ^]^ or the formation of oxidation^[^ ^47^, ^48^ ^]^ is irreversible, the above description in electrical properties strongly supports again that the doping/de‐doping reversibility is due to the adsorption/desorption processes of O_2_ molecules onto the surface of MoTe_2_. Other supplementary are provided in Figures S5 and S6, Supporting Information. Let us consider the energy‐band diagrams to explain the detail of the cycle (i.e., dry‐air and vacuum) of the RTA strategy for the MoTe_2_/SnS_2_ vdW heterostructure, as illustrated in Figure 4e. The adsorbed/desorbed O_2_ molecules act as carrier acceptors/donors to donate additional holes/electrons to the MoTe_2_ flakes, which results in its downward/upward *E*_F_ shift, as well as the polarity change of the vdW heterostructure. Additionally, notice that MoS_2_, the most used 2D material, is an intrinsically n‐type semiconducting channel because of the existence of sulfur vacancies and is relatively O_2_‐stable. It is widely believed that the charge polarity of MoS_2_ cannot be easily changed, compared with ambipolar‐type MoTe_2_. Therefore, the MoS_2_ potential is not considered in this work.

**Figure 5:**
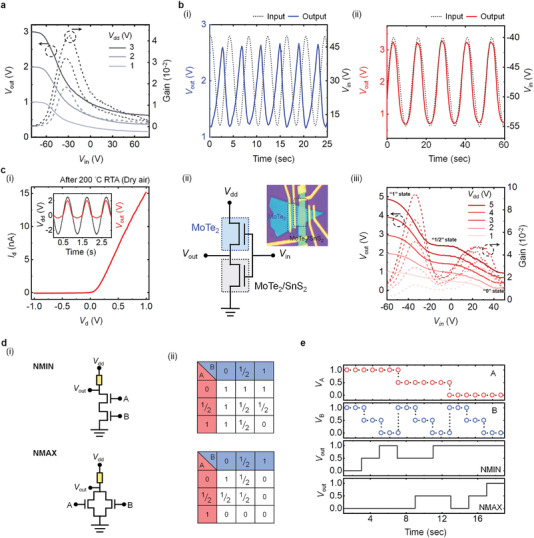
a) Transfer characteristics and voltage gains for an inverter operation, integrated by a unipolar MoTe_2_/SnS_2_ vdW heterostructure connected to a load resistor (1 GΩ). b) Oscillating signals of i) the common‐drain and ii) the common‐source mode measured at the output of an ambipolar MoTe_2_/SnS_2_ vdW heterostructure connected to a load resistor. ci) Output characteristics of diode operation at *V*_g_ = −60 V in an anti‐ambipolar MoTe_2_/SnS_2_ vdW heterostructure. The inset shows the features of a half‐wave rectifier. ii) Equivalent circuit for the ternary inverter and the corresponding OM image based on an anti‐ambipolar MoTe_2_/SnS_2_ vdW heterostructure connected to a MoTe_2_ transistor. iii) Transfer characteristics and voltage gains for the ternary inverter operation. d) Equivalent circuits and truth tables of NMIN and NMAX logic gates. e) Output voltages of NMIN and NMAX logic gates under applied input voltages (*V*_A_ and *V*_B_).

Integration of an O_2_‐ultrasensitive MoTe_2_ material and an O_2_‐insensitive SnS_2_ material to form a vdW heterostructure allows us to modulate and control the device under different degrees of oxygen adsorption. As proof of concept to multifunctionality in a single device, several operation modes of logic‐circuit applications were designed and demonstrated according to unipolar‐, ambipolar‐, or anti‐ambipolar‐mode MoTe_2_/SnS_2_ vdW heterostructure transistors, as illustrated in **Figure** **5**. First, an inverter function, which is one of the typical binary‐states circuits and can be normally realized in as‐fabricated MoTe_2_/SnS_2_ vdW transistors, was achieved using a unipolar vdW transistor connected to a load resistor. Figure 5a shows the transfer characteristics and the voltage gains (−d*V*_out_/d*V*_in_) of the inverter at different *V*_dd_ values, where *V*_out_, *V*_in_, and *V*_dd_ denote the output, input, and supply voltage, respectively. With a low *V*_in_ (i.e., logic 0), a high *V*_out_ (i.e., logic 1) is achieved, and vice versa. For the ambipolar‐mode feature, an OPC amplifier based on a single active device is presented. A sinusoidal signal *V*_in_ was applied to the gate electrode, while the output signal was detected using an oscilloscope. When the ambipolar MoTe_2_/SnS_2_ vdW transistor exhibited n‐type behavior, for larger/smaller *V*_in_, more conductive/insulating states, as well as smaller/larger *V*_out_, can be achieved, which is called the out‐of‐phase or common‐drain mode (see Figure 5bi). In contrast, when the vdW transistor worked as a p‐type channel, *V*_out_ increased synchronously with the increase of *V*_in_, which is called the in‐phase or common‐source mode for the OPC amplifier, as shown in Figure 5bii. The demonstration of both modes in the vdW transistors suggests that more complicated circuits could be developed such as phase‐shift key. The schemes of an inverter and OPC amplifier circuits equipped with an off‐chip resistor are shown in Figure S7, Supporting Information. The MoTe_2_/SnS_2_ vdW heterostructure transistor treated with the highest annealing treatment was further used to demonstrate the potential of the anti‐ambipolar mode feature. It is easy to understand as the vdW heterostructure is formed by a balanced p‐ and n‐transistor in series, in which the total current reaches a maximum when both transistors are turned on, and then the anti‐ambipolar behavior occurs. Therefore, a simple application using the anti‐ambipolar mode transistor is a rectifying p‐n diode, as shown in Figure 5ci. The inset displays the feature of half‐wave characteristics by applying sinusoidal *V*_dd_ at 5 Hz. Finally, we demonstrated a ternary‐states inverter, which is a basic block in multivalued logic applications. Figure 5cii shows the equivalent circuit of the vdW heterostructure‐based ternary inverter, which uses the MoTe_2_ transistor as a load resistor and the vdW heterostructure as a driver. The inset shows the corresponding optical microscopy (OM) image. Figure 5ciii plots the *V*_out_ variation and the voltage gains of the ternary inverter as a function of *V*_in_ at different *V*_dd_ values. When *V*_in_ was low, the vdW heterostructure region was fully turned off, but the MoTe_2_ transistor provided a small resistance path between *V*_dd_ and *V*_out_, leading to the output of logic “1.” When a large *V*_in_ was applied, the load resistor (i.e., MoTe_2_ transistor) was turned off, but the heterostructure reached a high conductance, and thus, the device outputs a logic “0.” At a moderate *V*_in_, the resistances of the load resistor and the driver were almost equal, which resulted in the output values of the logic state “1/2.” It is also observed that the width of the “1/2” state plateau can be controlled by applying *V*_dd_ values. The “1/2” logic state and its gain monotonically increase with *V*_dd_, which is attributed to how obvious is the anti‐ambipolar “Λ” shape in the transfer characteristics.^[^ ^19^ ^]^ To demonstrate the applicability of the ternary‐state devices for complex logic operations, NMIN and NMAX gates are further simulated. The logic circuit diagrams and the corresponding truth tables of the NMIN and NMAX are illustrated in Figure 5d, which are similar to AND and OR operations in the binary system, respectively. From Figure 5e, the transient responses of NMIN and NMAX are shown under variation of applied input voltages. The versatility described definitely demonstrates the great potential of our proposed RTA strategy for future device applications based on 2D vdW heterostructures.

## 2 Conclusion

In summary, we used a simple and effective RTA doping/de‐doping strategy under dry‐air and vacuum conditions, to realize a tunable and reversible charge‐polarity device based on MoTe_2_/SnS_2_ vdW heterostructures from unipolar, ambipolar to anti‐ambipolar behaviors. The core of the doping/de‐doping reversibility is responsible for the adsorption/desorption processes of O_2_ molecules onto the surface of MoTe_2_. Using the MoTe_2_/SnS_2_ vdW heterostructures, flexible multioperation functions, such as inverter, OPC amplifier, p‐n diode, and ternary‐state logic (NMIN and NMAX gates) were achieved, which provides a new doping strategy for layered electronics before integrated‐circuit packaging. Our work definitely demonstrates the potential for use in next‐generation electronics and will transcend the limits of Moore's law.

## 3 Experimental Section

### Device Fabrication

MoTe_2_/SnS_2_ vdW heterostructure transistors were fabricated by the dry‐transfer method. First, thin flakes of SnS_2_ were mechanically exfoliated from bulk SnS_2_ crystals onto a 300 nm thick SiO_2_ dielectric/Si substrate, while MoTe_2_ flakes were exfoliated from the bulk form onto a PDMS film (Gel‐Pak, PF‐40‐X4). The PDMS film attaching the exfoliated MoTe_2_ flakes was positioned on the arm of a micromanipulator and precisely transferred onto the top of the selected SnS_2_ flake. Subsequently, several pairs of contact electrodes were defined via conventional electron‐beam lithography. Ti/Au (20/60 nm) metal layers were then deposited at a base pressure of 2 × 10^−6^ torr, followed by a lift‐off approach in acetone to complete the device fabrication.

### Characterization

The morphology and thickness of the MoTe_2_/SnS_2_ vdW heterostructure were investigated using OM and AFM. Raman spectra were collected on a LabRam HR‐800 Raman spectrometer (Jobin Yvon), with a laser wavelength of 532 nm. Electrical measurements were performed under vacuum (<10^−5^ torr) in a cryogenic probe station (Lakeshore TTPX) with an Agilent B1500A semiconductor parameter analyzer.

## Conflict of Interest

The authors declare no conflict of interest.

## Supporting information

Supporting Information

## Data Availability

Research data are not shared.
