# Supplementary material for: Reversible Charge‐Polarity Control for Multioperation‐Mode Transistors Based on van der Waals Heterostructures
Source: Adv Sci (Weinh). 2022 Jul 13;9(24):2106016. doi: 10.1002/advs.202106016 (PMC9404391; doi:10.1002/advs.202106016)
Supplement: Supplementary file 1 — Supporting Information [file ADVS-9-2106016-s001.pdf]

ADVANCED SCIENCE
Open Access

Supporting Information

for *Adv. Sci.*, DOI 10.1002/advs.202106016

Reversible Charge-Polarity Control for Multioperation-Mode Transistors Based on van der Waals Heterostructures

Ciao-Fen Chen, Shih-Hsien Yang, Che-Yi Lin, Mu-Pai Lee, Meng-Yu Tsai, Feng-Shou Yang, Yuan-Ming Chang, Mengjiao Li, Ko-Chun Lee, Keiji Ueno, Yumeng Shi*, Chen-Hsin Lien, Wen-Wei Wu, Po-Wen Chiu, Wenwu Li*, Shun-Tsung Lo and Yen-Fu Lin**

Supporting Information

Reversible Charge-polarity Control for Multioperation-mode Transistors Based on van der Waals Heterostructures

Ciao-Fen Chen[#], Shih-Hsien Yang[#], Che-Yi Lin, Mu-Pai Lee, Meng-Yu Tsai, Feng-Shou Yang, Yuan-Ming Chang, Mengjiao Li, Ko-Chun Lee, Keiji Ueno, Yumeng Shi*, Chen-Hsin Lien, Wen-Wei Wu, Po-Wen Chiu, Wenwu Li*, Shun-Tsung Lo, and Yen-Fu Lin**

C. F. Chen, Prof. S. T. Lo
Department of Electrophysics and Center for Emergent Functional Matter Science (CEFMS), National Yang Ming Chiao Tung University, Hsinchu 30010, Taiwan

Dr. S. H. Yang, Prof. Y. Shi
International Collaborative Laboratory of 2D Materials for Optoelectronics Science and Technology (Ministry of Education), Engineering Technology Research Center for 2D Material Information Functional Devices and Systems (Guangdong Province), Institute of Microscale Optoelectronics, Shenzhen University, Shenzhen 518060, China
E-mail: yumeng.shi@szu.edu.cn

M. P. Lee, Prof. W. W. Wu
Department of Materials Science and Engineering, National Yang Ming Chiao Tung University, Hsinchu 300, Taiwan

Prof. W. W. Wu
Center for the Intelligent Semiconductor Nano-system Technology Research, National Yang Ming Chiao Tung University, Hsinchu 300, Taiwan

M. Y. Tsai, F. S. Yang, Dr. K. C. Lee, Prof. C. H. Lien, Prof. P. W. Chiu
Institute of Electronics Engineering, National Tsing Hua University, Hsinchu, 30013, Taiwan

Prof. K. Ueno
Department of Chemistry, Graduate School of Science and Engineering, Saitama University, Saitama 338-8570, Japan

Prof. Wenwu Li
Shanghai Frontiers Science Research Base of Intelligent Optoelectronics and Perception, Institute of Optoelectronics, Department of Materials Science, Fudan University, Shanghai 200433, China
Email: liwenwu@fudan.edu.cn

C. F. Chen, Dr. S. H. Yang, Dr. C. Y. Lin, M. P. Lee, M. Y. Tsai, F. S. Yang, Dr. Y. M. Chang, Dr. M. Li, Prof. Y. F. Lin
Department of Physics, National Chung Hsing University, Taichung, 40227, Taiwan
E-mail: joe801105@gmail.com, yenfulin@nchu.edu.tw

Prof. Y. F. Lin

Department of Materials Science and Engineering, Institute of Nanoscience, *i*-Center for Advanced Science and Technology (*i*-CAST), National Chung Hsing University, Taichung, 40227, Taiwan

[#]Ciao-Fen Chen and Shih-Hsien Yang contributed equally to this work.

Keywords: $MoTe_2$, SnS_2, van der Waals heterostructures, multioperation-mode transistors, charge-polarity control

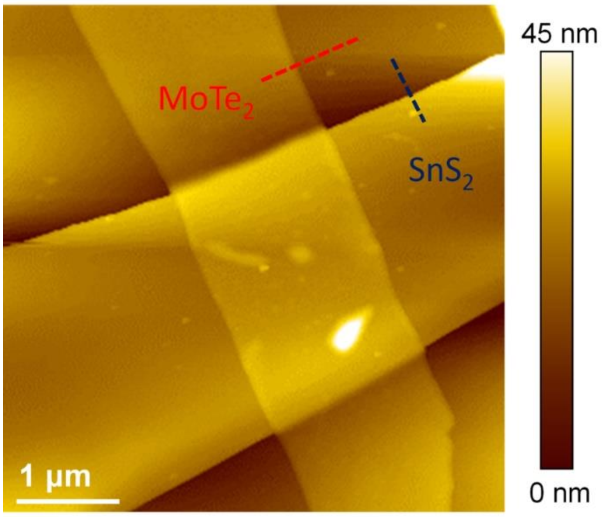

Figure S1. AFM image of a $MoTe_2/SnS_2$ heterostructure deposited on a silicon substrate with a 300-nm thick SiO_2 layer. The dashed red and black lines indicate the cross-sectional profiles for the $MoTe_2$ and SnS_2 flakes, respectively, as shown in Figure 1b.

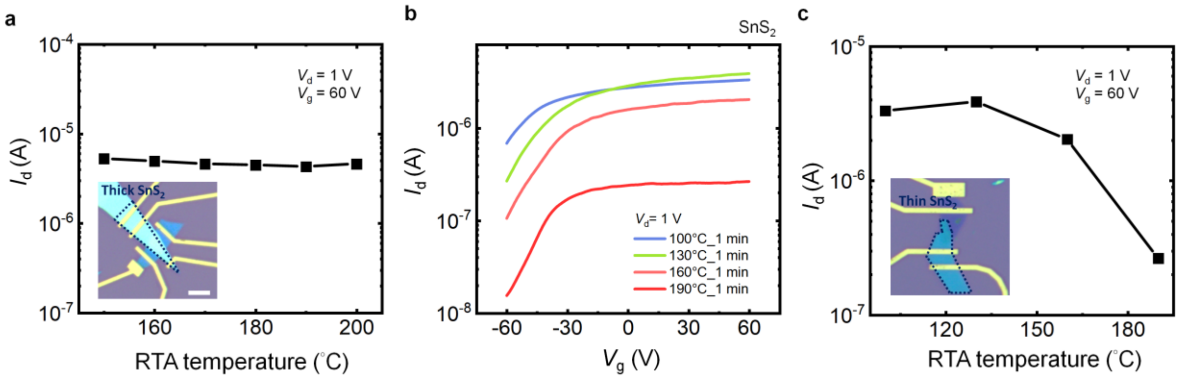

Figure S2. (a) The source-drain current at V_g= 60 V for the thick SnS_2 transistor (shown in Figure 3, main text) at different annealing temperatures from 150 °C to 200 °C under dry-air conditions during the RTA process. The inset in (a) shows the optical device image, where the SnS_2 thickness is approximately 30 nm. (b) Transfer characteristic curves and (c) the corresponding source-drain current at V_g= 60 V for a thin SnS_2 transistor at different annealing temperatures from 150 °C to 190 °C in dry-air conditions during the RTA process. The inset in (c) shows the optical device image, where the SnS_2 thickness is approximately 5 nm.

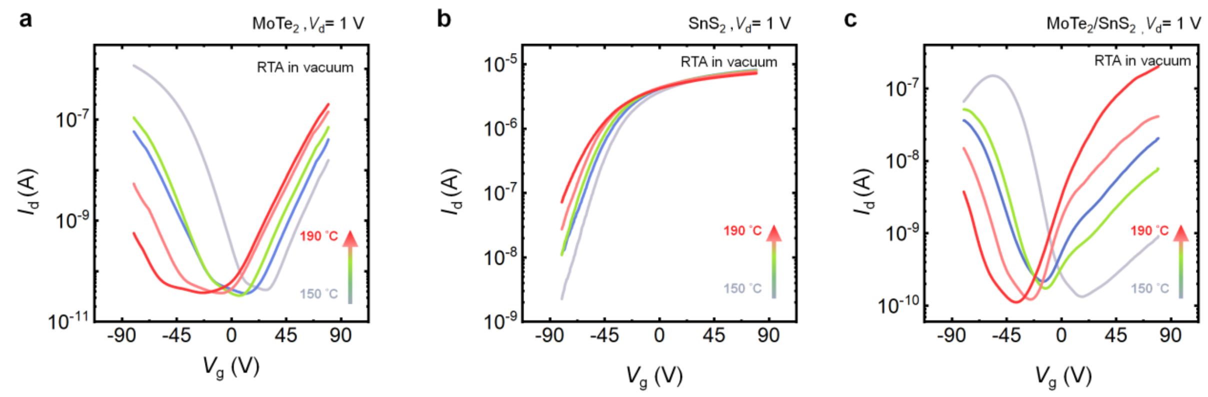

Figure S3. Transfer characteristic curves of another (a) $MoTe_2$ and (b) SnS_2, and (c) $MoTe_2/SnS_2$ heterostructure in semilog scale. The different color curves represent different annealing temperatures from 150 °C to 190 °C in vacuum conditions during the RTA process.

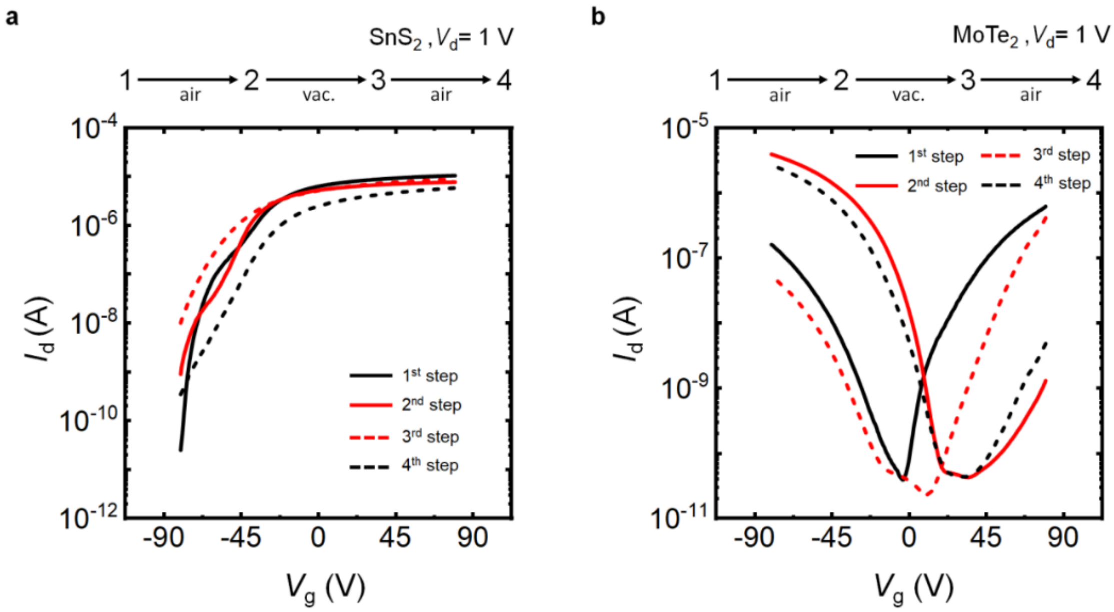

Figure S4. Reversibility of the RTA process, including cyclic exposure to dry air and vacuum conditions, for the (a) SnS_2 and (b) $MoTe_2$ transistors. Transfer properties before the RTA process (first step, solid black line). Transfer properties of the heterostructure processed using the RTA process in dry air (second step, solid red line). Transfer features of the heterostructure after the RTA process in a vacuum (third step, dashed red line) and dry air (fourth step, dashed black line).

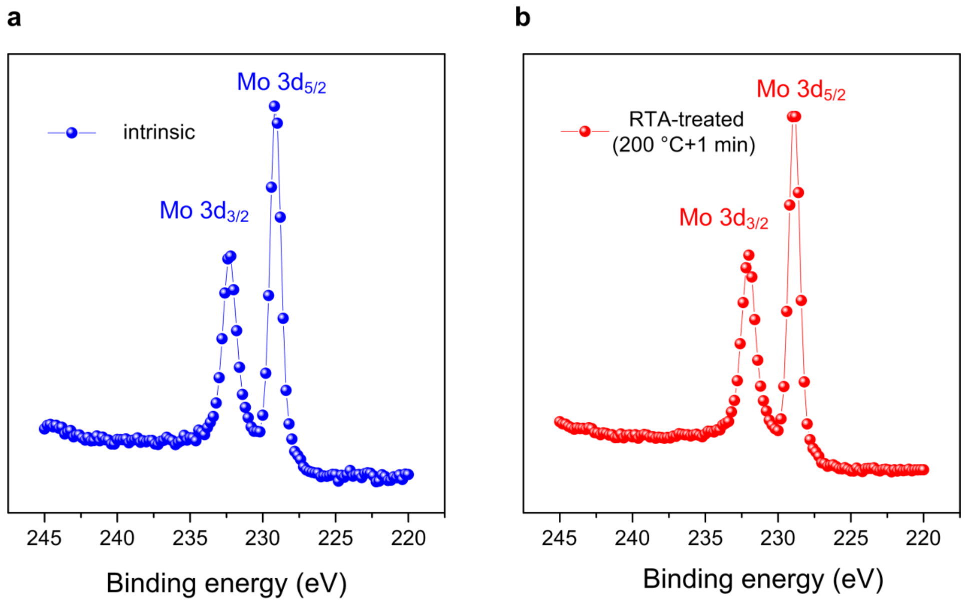

Figure S5. XPS characteristics of the $MoTe_2$ transistor (a) before and (b) after the RTA process.

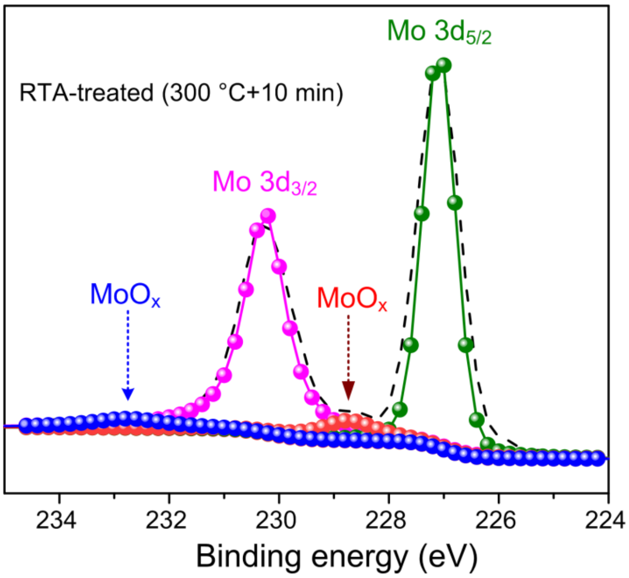

Figure S6. XPS characteristics of the $MoTe_2$ transistor after the RTA process with a higher temperature (300 °C) and longer retention time (10 min).

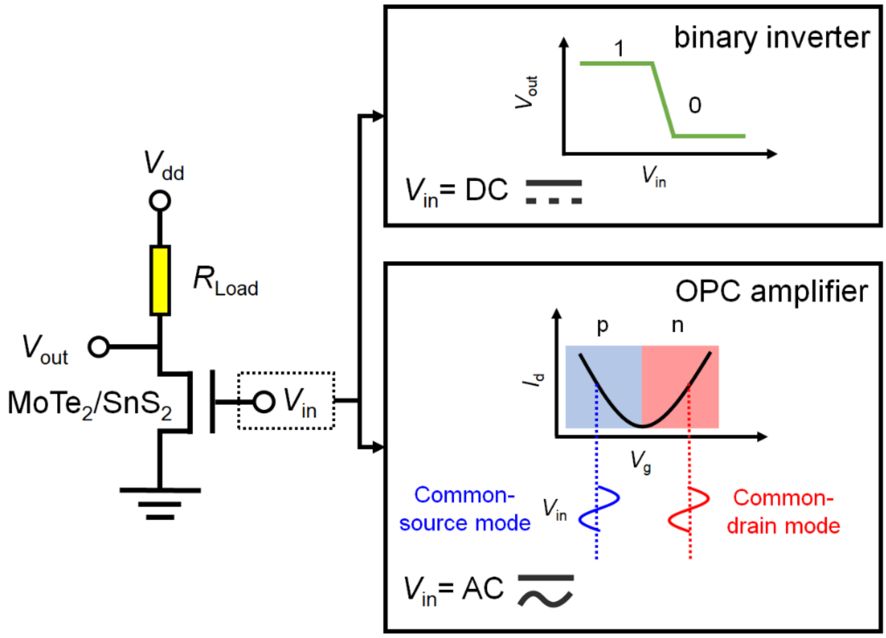

Figure S7. Schemes of an inverter and OPC amplifier circuits equipped with an off-chip resistor.
